# Patient and clinician acceptability of an integrated physiotherapy and nutrition intervention after ICU discharge: a qualitative exploration of a pre-specified co-primary feasibility outcome of the PHOENIX trial

**DOI:** 10.1016/j.eclinm.2026.104085

**Published:** 2026-07-20

**Authors:** Elizabeth King, Nicola Wyer, Dalia Barghouthy, Ellen Reynolds, Holly Richardson, Miles Negus-Fancey, Louise Gallie, Zudin Puthucheary, Owen Gustafson, David McWilliams

**Affiliations:** a Therapies Clinical Service Unit, Oxford University Hospitals NHS Foundation Trust, Oxford, UK; b NIHR Blood and Transplant Research Unit, University of Oxford, Oxford, UK; c University Hospitals Coventry & Warwickshire NHS Trust, Coventry, UK; d Patient Representative, UK; e William Harvey Research Institute, Queen Mary University of London, London, UK; f The Royal London Hospital, Barts Health NHS Trust, Whitechapel Road, London, UK; g Centre for Care Excellence, Coventry University, Coventry, UK

**Keywords:** Intensive care unit, Rehabilitation, Nutrition, Critical illness, Survivorship

## Abstract

**Background:**

Recovery after critical illness is complex and multi-faceted. There are widespread variations of rehabilitation interventions delivered when patients step-down onto the ward. The aim of the study was to evaluate the acceptability of a structured, individualised physiotherapy and nutritional intervention delivered to patients following discharge from intensive care, from the perspectives of both patients and healthcare professionals.

**Methods:**

This qualitative study was embedded within a two-centre randomised controlled feasibility trial of an enhanced physiotherapy and optimised nutrition intervention delivered in the ward setting after ICU discharge. It evaluated acceptability, a pre-specified co-primary feasibility outcome of the trial. Between May 2024 and January 2025, patients stepped down from ICU to ward based care, alongside healthcare professionals involved in or affected by intervention delivery were invited to participate. Semi-structured interviews were conducted, audio recorded, transcribed verbatim, and analysed using reflexive thematic analysis. Themes were then mapped across the constructs of the Theoretical Framework of Acceptability (TFA). The trial was prospectively registered (ClinicalTrials.govNCT06159868).

**Findings:**

Eleven patients and eight healthcare professionals participated in interviews. Three overarching themes were identified. *‘Increased awareness of combined nutrition and physical recovery’* focused on recognition of the complementary nature of nutritional intake and rehabilitation progress. The second theme–‘*Accurate nutritional goals to enable focused rehab’* emphasised the need for individualised nutritional prescriptions to underpin rehabilitation. The final theme–*‘Supporting patient recovery to restore independence and purpose’* highlighted the need for a holistic recovery following critical illness. These themes were mapped to six of the seven TFA constructs.

**Interpretation:**

Both patients and staff perceived the integrated physiotherapy and nutrition delivered in the ward setting as acceptable.

**Funding:**

This project is funded by the 10.13039/501100000272National Institute for Health and Care Research (NIHR) under its Research for Patient Benefit (RfPB) Programme (Grant Reference Number NIHR205370).

**Research in context**

**Evidence before this study**

This paper importantly reports the acceptability of an integrated physiotherapy and nutrition intervention after ICU discharge. The paper is written with the findings introduced with the themes following thematic analysis and then mapped according to the Theoretical Framework of Acceptability constructs (achieving six out of the seven constructs). In particular, this qualitative paper achieves one of the co-primary feasibility outcomes of the McWilliams D, Gustafson O, Wyer N, et al. Physiotherapy and Optimised Enteral Nutrition In the post-acute phase of critical illness (PHOENIX): a randomised controlled feasibility trial. 2026. eClinicalMedicine. (In Press). The acceptability findings included improved awareness and education for staff, engagement through therapeutic relationships, perceived value in the accuracy of measuring nutrition, and both interventions contributing to progressive recovery from critical illness. The study intervention was performed in the ward setting (post ICU) and this time point of acceptability is novel.

**Added value of this study**

The essence of therapeutic relationships to support recoveries, integrated working between professional groups, education and use of the indirect calorimetry align with previous research, albeit in different patient groups.

**Implications of all the available evidence**

The findings from this interview study embedded within a feasibility RCT support evidence for a full trial of the intervention. In the meantime, the above findings are value to be translated into clinical practice to support minimising professional silos, improved education for patients and staff, acceptability with use of the indirect calorimetry and focus on recovery from critical illness.

## Introduction

Survival following an admission to an intensive care unit (ICU) is increasing, despite patients presenting with greater multimorbidity and frailty.^1^ Recovery after critical illness involves multiple milestones with the ultimate goal to restore the patients function and independence. Transition from ICU to the ward represents a critical stage in the recovery trajectory with 98% of patients require ongoing physiotherapy and 70% are at risk of malnutrition.^2^ Despite these outstanding rehabilitation needs, there are widespread variations in ward-level rehabilitation delivery across United Kingdom (UK) hospitals.^1^ Following discharge from ICU, access to therapeutic interventions from the multidisciplinary team are frequently reduced, contributing to ongoing physical deconditioning and malnutrition. This results in over a half of patients experiencing a deterioration in physical status on step-down to the ward environment, particularly affecting those who leave the ICU most debilitated. Malnutrition from ICU often persists and can worsen on the ward with multiple identified barriers to achieving adequate nutritional.^3^^,^^4^ Physical deconditioning and malnutrition are known to contribute to poor patient outcomes and result in barriers to rehabilitation.

Qualitative literature exploring recovery following critical illness has largely centred on physical rehabilitation in and after ICU, focusing on engagement and patients' vulnerability as they transition back into the community.^5^ Nutritional challenges including reduced appetite, altered taste, gastrointestinal symptoms, and changes in physical appearance can further impede meaningful recovery.^6^ Despite this, physical and nutritional recovery often remains poorly supported beyond hospital discharge.^7^ To date, few studies have explored how integrated rehabilitation and nutritional interventions are experienced by patients and healthcare professionals during the ward-based phase of recovery. Interventions have included nursing staff education, and implementation of nutritional support and ambulation which were well received by nursing staff, but patient's adherence reduced when they were expected to act on their own.^8^^,^^9^ The exploration of such interventions are required in the post-ICU population given their acuity including muscle catabolism^10^

Evaluating acceptability is a key component in the design and evaluation of healthcare delivery within the context of developing a complex intervention. Interventions that are considered acceptable are more likely to be implemented effectively, adhered to, and sustained in practice.^9^^,^^11^^,^^12^ Aligned to the Theoretical Framework of Acceptability (TFA).^13^ The aim was to evaluate the acceptability of structured, individualised physiotherapy and nutritional intervention delivered to patients following discharge from intensive care, from the perspectives of both patients and healthcare professionals.

## Methods

### Study design

This qualitative evaluation of acceptability was embedded within a two-centre, parallel-group, randomised controlled feasibility trial.^9^ The trial was prospectively registered (ClinicalTrials.gov NCT06159868). The intervention comprised structured, individualised physiotherapy combined with optimised nutrition delivered following step-down from ICU to the ward for up to 14-days or hospital discharge, whichever occurred sooner. A detailed description of the PHOENIX intervention in accordance with the Template for Intervention Description and Replication (TIDieR) checklist is provided in the Supplementary Material 1. The intervention was developed in line with Medical Research Council guidance for complex interventions and informed by existing evidence, multidisciplinary expertise, and iterative refinement. Detailed information regarding the development, rationale, and implementation of the PHOENIX intervention is described in the open access published trial protocol.^14^ Participants allocated to the control group received usual ward-based care, including standard physiotherapy, dietetic input, and multidisciplinary rehabilitation as determined by the treating clinical teams at each site, This qualitative study reports the acceptability of the intervention to participants and service providers, which was prespecified as a co-primary feasibility outcome in the PHOENIX trial protocol.^14^ Quantitative feasibility outcomes (including recruitment, retention, and intervention adherence) are reported separately.^15^ The COREQ (Consolidation Criteria for Reporting Qualitative Research) reporting guideline checklist^15^ was used for this manuscript preparation (Supplementary Material 2).

The TFA allows for evaluation of intervention acceptability based on lived or perceived experiences by those delivering or receiving the intervention. It accounts for personal, organisational and contextual factors that might influence the delivery and acceptability through the seven constructs.

### Ethics

The study was approved by the Wales Research Ethics Committee 2 (24/WA/0050). Written informed consent was obtained from participants.

### Participants

The study took place within two university teaching hospitals in the UK. All patients recruited to the study and randomised to the intervention group were eligible to take part in the interviews. Patient participants were purposively sampled to ensure an appropriate mix of age, gender and reason for admission. Staff participants were eligible to be invited to interview if they were directly involved in delivery of the research intervention or directly affected by the additional physiotherapy and/or nutritional interventions. Staff criteria for purposive sampling included a range of professions, wards and different study engagement. For both participant groups, if interest in being interviewed was expressed, a participant information sheet was provided with suitable time to consider their involvement prior to the interview. Suitability for interview participation, including ability to communicate effectively in English, was assessed during read back and recall through recruitment discussions and the consent process.

### Data collection

Patients were interviewed face to face prior to hospital discharge, where reasonably practical, or shortly after hospital discharge via telephone or face to face at their homes (by EK/DB and ER). Staff were interviewed face to face at any time during, or shortly after the intervention period at their hospital site. All participants gave written consent prior to interview, and were advised they could withdraw at any time, without giving a reason.

Semi-structured interviews followed a topic guide which was developed by the research team allowing for flexible exploration of participant experiences beyond the initial questions. Patient and staff interviews explored their experiences of receiving or delivering the intervention, barriers to engagement or delivery, and ideas for future improvement (Supplementary Materials 3 and 4. Staff interviews also explored the evaluation of care co-ordination between the specialist rehabilitation team and the usual ward teams. All interviews were audio recorded and transcribed verbatim. Data saturation was achieved when sufficient data to identify themes had been established, no new meanings emerged, and data sufficiently answered the research question.^16^

### Data analysis

Data were analysed using reflexive thematical analysis following the six steps of Braun and Clarke.^17^ This was an inductive approach, whereby themes were developed from codes derived from patterns and meanings within the dataset. Step one was data familiarisation where researchers (EK, NW, DB and ER) read the interview transcripts and aimed to immerse themselves in the data with note making of initial patterns. Step two was coding the data where the researchers (EK, NW, DB and ER) developed preliminary codes. Patient and staff codes were initially separated to explore differences and then combined by constant comparison for similarities and differences. Step three involved generation of the initial themes, where researchers (EK, NW, DB and ER) collaboratively developed preliminary themes. This was followed by step four, where themes were reviewed and developed by discussion across the research team. Step five refined the themes, including revisiting the coding books and transcripts, and the use of mind maps to define the themes and their essence. Through an abductive approach, themes were then mapped against the constructs of the TFA. The final step was reporting of the findings, including refinements of theme descriptions led by EK and NW with discussion and feedback from DB and ER. The final themes were presented to two patient public involvement and engagement representatives from the trial management committee, who were prior patients with prolonged ICU admissions. They agreed the themes resonated with their experiences and supported the language used within theme titles. Themes were refined through iterative discussion across the research team. In keeping with reflexive thematic analysis, the purpose of these discussions was not to achieve statistical consensus, but to support interpretive exploration and development of shared analytical understanding.

### Rigor and reflexivity

The research team comprised experienced qualitative researchers and health care professionals working in critical care and rehabilitation settings. EK and DB are physiotherapists with clinical roles in ICU recovery pathways, while NW and ER are dietitians with expertise in nutrition support across the critical care continuum. The team's professional backgrounds provided contextual understanding of post-ICU rehabilitation and nutrition; however, this positioning also had the potential to shape data generation and interpretation. In addition, some patient participants were known to the research team from ICU or the wards, and some of the staff participants had working relationships with them.

Potential research bias was considered throughout the study, recognising that the professional backgrounds and clinical experiences of the research team could influence data interpretation. Reflexive discussions were undertaken throughout analysis to enhance awareness of pre-conceived assumptions, support challenge and discussion of interpretation, and strengthen trustworthiness. This also included writing research memos during data collection and analysis to consider their confirmability.^18^ Findings were grounded in participant accounts through iterative review of transcripts and use of verbatim quotations. The analysis development was guided by EK and NW who have substantial experience undertaking qualitative research.

### Role of the funding source

This project is funded by the National Institute for Health and Care Research (NIHR) under its Research for Patient Benefit (RfPB) Programme (Grant Reference Number NIHR205370). The funder had no role in study design, data collection, data analysis, data interpretation, or writing of the report. The views expressed are those of the authors and not necessarily those of the NIHR or the Department of Health and Social Care.

## Results

**Fig. 1:**
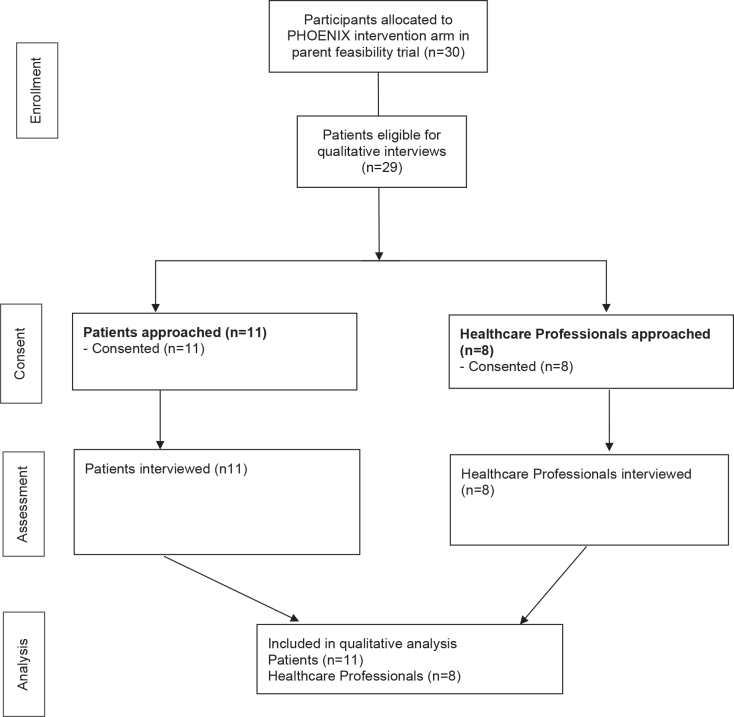
Participant flow diagram for the qualitative evaluation embedded within the PHOENIX feasibility trial.

The wider PHOENIX trial [15 recruited 60 patient participants across both sites, of which 30 were randomised to the intervention group (see Fig. 1), including six who received the indirect calorimetry. Interviews were undertaken with 11 patient participants and eight staff participants, recruited from across both research sites. All participants approached were consented.

Seventy six percent of the patient population were female, with a median age of 63 years and a median ICU length of stay (LOS) of 16 days (Table 1). All staff participants were female, with six physiotherapists, one dietitian and one therapy support worker. The mean duration of the interviews was 17.5 min (range 5–28 min) across both groups.

**Table 1:** Patient participant demographics.

Participant number	Gender	Ethnicity	Age	Reason for admission	ICU LOS
A1	Male	Asian/British Asian–Indian	49	Surgery	16
A2	Male	White British	75	Trauma	13
A3	Male	White British	75	Surgery	15
A4	Male	White British	71	Surgery	24
A5	Male	White British	42	Trauma	16
A6	Female	White British	76	Neuro	23
B1	Male	White British	57	Respiratory (Non COVID)	5
B2	Female	White British	76	Respiratory (Non COVID)	36
B3	Male	White British	59	Respiratory (Non COVID)	11
B4	Female	White British	63	Respiratory (Non COVID)	12
B5	Male	White British	43	Respiratory (Non COVID)	4

Three overarching themes were developed: *‘Increased awareness of combined nutrition and physical recovery’* which focused on the complementary nature of these therapeutic interventions; ‘*Accurate nutritional goals to enable focused rehab’* which emphasised the recognition of underpinning prescriptions; and ‘Supporting *patient recovery to restore independence and purpose’* which highlighted the need for a holistic recovery following critical illness. The three themes were mapped across six of the seven TFA constructs (Table 2). The findings are reported under the constructs of the TFA with illustrative quotes.

**Table 2:** Summary of findings from the themes mapped to the TFA.

Theme	TFA construct	Quotes
Increased awareness of combined nutrition and physical recovery	Affective attitude	*BS3: “Yeah, I guess it's a bit of a learning need for both teams … for us [physiotherapists] to maybe reach out and get a bit more information about if there's anything from our perspective that we might be able to highlight to dietitians … I guess how much we're doing, what we might be limited by, and maybe reaching out and learning a bit more about that, because I guess the communication between the two is probably quite key.” (physiotherapist)* B2: *“Well, I quite enjoyed the ball games because we had a laugh while we were doing it. And it kind of kind of tightened up my stomach muscles and white little muscles I've got left in my arms. You are catching and moving as well”*. *B4: “I didn't realise how being in intensive care could make you. It was just like my legs wouldn't do what my body wanted them to do”. (patient)* *AS2: “And it made me just realise there's probably other patients out there that on face value “Oh, he's eating great. Discharge him,” but we only picked him up because he was in the trial.” (dietitian)*
	Perceived effectiveness	*AS1:* “*To be honest, not until the Phoenix trial … [I knew] it was important, but you didn't realise how much it [nutrition] would affect their rehab until we did this trial. How it links in. It's given us more awareness. With their exercise, they need their nutrition to be able to do stuff, they need the regular weights as well*.*” (therapy support worker)* *BS1: ‘It's probably something that I should do more in, but it's not something that I tend to routinely get involved with. I mean I'll have an awareness if they're on a feed or they're drinking the Ensures … but it's probably something I don't routinely ask about.” (physiotherapist)* *AS2: “… they just see them when they step down, and everything that's happened before that is perhaps a little bit of a mystery because they don't have the knowledge of what ventilators are and all the medications they might have been on, and how much weight they lose in that time” (dietitian)*
	Self-efficacy	*BS4: “Yeah, I think it allows for learning on both sides … So I think from an NIV perspective … it's our kind of specialist area, yet when patients are coming acutely from ITU and still on maybe 50–60% on the high flow and still quite unwell, we value the support that we get from the ITU team to say, this is kind of where we were at downstairs on the unit, this is where we were thinking of going and can you support us, … It's just having that extra support in an acute manner.” (physiotherapist)*
Accurate nutritional goals to enable focused rehab	Affective attitude	*AS2: “If you use the equations that we're taught to use, then you would under feed him. If you have the indirect calorimetry, you can actually measure that. So I think that's the other benefit from the trial is we're measuring what they actually need, not what we think they need based on potentially sketchy evidence” (dietitian)* *A4: “Up to now, I was losing weight. When I got that [indirect calorimetry], I've put on about six pounds in ten days” (patient)*
	Burden	*A1: “I was quite happy for the process to happen. I think they did try to do it first. Then, for some reason, I kept setting off the machines” (patient)* A1: *“I was happy to help conduct that as well because some people can get claustrophobic in that. I will say it's like an MRI scan. You have to take your mind away. You have to close your eyes, take your mind away and just do what you need to do. Then, that's it” (patient)*
	Perceived effectiveness	*AS2: “Some of these guys need 60 or 70 calories per kilogram. So if you use the equations that we're taught to use, then you would under feed him. If you have the indirect calorimetry, you can actually measure that.” (dietitian)* *BS1: “if you get their nutrition on board, you're giving them more energy to be able to then rehab or fight infection … You can hopefully be giving them those calories and that energy and then they've got more energy to rehab and do the exercises.” (physiotherapist)*
	Intervention coherence	*AS3: “Sometimes you find that people may not get enough. They don't build as much strength, or they don't improve as much as you would expect them to, and it might be because they're not getting what they need to out of their feed either” (physiotherapist)* *AS1: “If they're not getting the proper nutrition, we're not going to get anywhere, to be honest. They're weak, they're fatigued, and also they're in lower mood because they're hungry” (therapy support worker)*
Supporting patient recovery to restore independence and purpose	Ethicality	*A1: “… it was learning how to walk again, learning how to chew food, eat, swallow, drink. You don't think about it and then, once you do, it's like oh. It's like a little achievement, goal”. (patient)* *B1: “And sort of like two a day [ensures} was enough for me. But I kind of like kept to it because I wanted to sort of come home. So, I kind of like kept to it. So, if I didn't eat a meal, they would obviously monitor that and then the next thing the Ensure would appear.” (patient)* *AS2: “I think it's the patient outcomes that we're thinking about. So some of the basic ones might be things like preventing them from losing more weight, keeping at a healthy BMI … so that they can get back to their normal life quicker, so they can get home and be fit and well and active and resume work …”*. *(dietitian)*
	Perceived effectiveness	*A3: “Well, the lady came in daily and advised … She was asking me what did I have for lunch, what did I have yesterday, what did I have for dinner? … She put me on various supplements, which were all designed to boost my strength. I felt, quite honestly, she was giving good advice, told me what sort of food I need to be eating … I used to look forward to her coming because she gave really good, sound advice”. (patient)*
	Intervention coherence	*AS4: “if a person is not having any nutrition, then for me, every other thing we're doing is probably futile … Now, imagine somebody who has been very sick, who has loads of medications being pumped into their system, without food … So, I personally think it's at the top of our list of things to look at when they're treating patients …” (physiotherapist)*
	Self-efficacy	*B1: “Well, to start with it made me feel I don't know really, a bit ashamed … I've always been sort of like able to get over things. But with panicking that was all kind of new. I think she knew it was kind of new so, like I said, we had a number scale that if we got to six and over that was the danger. That was the time to, like, stop, take stock … And just breathe.” (patient)* *A3: “It (physio) was well planned. It was well thought of. They didn't put too much pressure on you, which helped, when you're trying to basically, learn to walk again”. (patient)* *B2: “So, I was quite motivated to sort of do as much as I could, but the physio was reminding me to slow down a bit and not to go too fast … It's been a sort of developmental evolutionary process as strength and energy have developed.” (patient)*

### Affective attitude

Patient and staff participants expressed positive feelings towards the delivery of the combined nutritional and physiotherapy interventions to support patient recovery from critical illness. Patients expressed the impact of their critical illness including fatigue and weakness (B4 in Table 2) Patients reported enjoying the regularity of intervention, cited often daily for physiotherapy but less frequently for dietitians. They were also grateful for the diversity of interventions such as education, mobility, games and exercise, and going off the ward (B2 in Table 2).

Dietitians felt the trial was a vehicle to support them in identifying the nutritional needs of patients through direct clinical contact at the bedside (AS2 in Table 2) This contrasts with previous reliance on accepting verbal handovers that patients had returned to eating independently. Staff perceived this contact to facilitate independent clinical reasoning to direct treatments rather the risking potential missed opportunities to intervene. Staff also perceived the direct contact with patients to facilitate conversations and enabled more individualised understanding of patient needs. This then directly guided the frequency of clinical reviews or triggered an increase in subsequent interventions, suggesting clinical flexibility and responsiveness to patient need.

Both physiotherapists and dietitians reflected how the study allowed them to consider how the constructs of nutrition and physical rehabilitation could align and complement each other to support ongoing recovery from critical illness. Staff identified the value of understanding what patients were doing as part of their rehabilitation to recognise and modify their nutritional needs, whilst also understanding their current nutritional needs and targets for optimal intake. Staff described how the use of indirect calorimetry provided an objective measurement of calorie requirements compared with the limitations of predictive equations routinely used in standard care: This suggests that an increased knowledge of clinical status and current needs has the potential to guide and modify both nutritional intake and rehabilitation interventions to optimise response.

There was a consensus that physiotherapists and dietitians could increase their collaborative working enabling the delivery of integrated therapies, underpinned by improved communication (BS3 in Table 2). It was perceived staff would need open channels of communication, and this would enhance learning for both specialities to improve patient care. This suggests the importance of integrated therapies to deliver optimal patient care for recovery, rather than being limited by professional silos.

### Burden

Perceptions of burden were only recognised by patients. One patient referred to alarms during the calorimetry use (B1 in Table 2). Whilst not reported by those who took part in the study, one patient suggested that the clear plastic canopy hood used during measurement could potentially make people feel claustrophobic (B1 in Table 2).

### Ethicality

Ethicality centred about the hope for recovery and restoring fundamental activities of daily living. Patients recognised how far they were off their baselines of mobility, function and independence. Gradual progression in abilities and such improvements were celebrated by one patient (A1 in Table 2). Adherence to nutrition was a catalyst for being safe to return home (B1in Table 2), regarded as a key milestone by one patient. Staff also recognised patient dependency following critical illness, identified how patient outcomes extend beyond hospital discharge to returning to being well and returning to work (AS2 in Table 2). This framed the trajectory of their recovery following critical illness as an entity.

### Perceived effectiveness

Perceived effectiveness was found in all of the three themes. The intervention was perceived to increase professional awareness as staff stepped out of their clinical silos. Staff also perceived the need to understand patient's nutritional status as valuable to guide rehabilitation and the study facilitated this. In particular, optimised nutrition, strength and wider recovery such as fighting infections. Patients (A3 in Table 2) valued the education from the dietitian leading to a positive patient experience. The delivery of meaningful and individualised interventions suggests patient centred care is essential despite the underpinning of constrained and dynamically changing healthcare systems.

### Intervention coherence

A physiotherapist highlighted that nutrition is an essential component to patient recovery and it's absence, interventions are potentially futile suggesting it (AS4 in Table 2). From a dietetic perspective, accurate assessments were perceived to minimise the risk of over or underfeeding whilst also allowing sufficient energy (AS1 & AS2 in Table 2). Both patients and staff recognised how the integrated intervention is facilities patient recovery, in particular with modifications and progressions based on clinical needs.

### Self—efficacy

Patients and staff expressed self-efficacy with similarities of receiving respective support. Ward physiotherapists valued shared working and to learn from ICU colleagues who knew the patient's history. Most patients expressed their gratitude of such positive therapeutic relationship with staff. Patient confidence to engage in their rehabilitation was supported through pacing, reassurance and encouragement (A3 & B1 in Table 2) These supporting factors combined suggest the potential for partnerships in care, contributions to informed patient decision-making and attainable recovery.

## Discussion

This qualitative evaluation, embedded within a feasibility RCT, explored the acceptability of structured, individualised physiotherapy and optimised nutrition for patients following step-down from ICU to the ward.

The findings have been mapped to the linear nature of the TFA constructs. However, several findings were interconnected in keeping with real-world healthcare practices. To our knowledge, this is the first qualitative exploration following physiotherapy and dietetics interventions following admission to ICU. Patients described how the interventions were well received and contributed to their recovery following critical illness. Patient benefits included positive experiences of progressive physical rehabilitation, and for those who received indirect calorimetry, perceptions of value from accurate nutritional assessments. Staff described how the study increased their awareness of integrated therapies, shared expertise and communication to understand and respond to patient needs.

Recovery and rehabilitation after critical illness is multifaceted and complex due to the individual needs of the patient. Restoring physical function is often regarded as a priority in the patient journey contributing to their ability to return home and renew their independence.^19^^,^^20^ Whilst nutrition is known to play a role in aspects of life such as well-being, quality of life and general health, it is often not readily offered as a priority.^21^^,^^22^ In this study, patients recognised that they needed to relearn to chew, swallow and eat again after their critical illness and their nutrition could lead to strength and hospital discharge. Therefore, this suggests a signal that the patients appeared to increasingly recognise nutrition as an active and important component of recovery. This greater awareness and engagement with nutrition during admission may have implications for ongoing nutritional behaviours and recovery following hospital discharge.

This study aligns with other qualitative research where integrated therapies from both physiotherapists and dietitians are an ‘eye opener’ to increase awareness of the multifaceted health interventions rather than siloed working.^23^ In another study, physiotherapists and dietitians are considered to be advocates for interprofessional collaborations within the remit of malnutrition and sarcopenia.^24^ Beyond collaborative working, Reinders et al., recognised the need to increase interprofessional knowledge.^24^ Previously physiotherapists working with patients with chronic conditions expressed low confidence in their knowledge and skills of nutrition care, and to improve such integration within routine practice they would benefit from education on immune function, metabolism and functional performance.^25^ Similarly in this study, both professions suggested a willingness to recognise, learn and adapt their practice to enhance the holistic nature of patient interventions.

The use of indirect calorimetry is considered the gold standard for measuring resting energy expenditure assessment.^26^ However, limited evidence exists regarding its use in acute clinical practice by healthcare professionals, or the experience of patients themselves. Muller et al. conducted a study in trauma patients to determine the feasibility and acceptability of indirect calorimetry, out of 30 measurements two were not completed due to patient discomfort or pain.^27^ In those patients who completed a post procedure survey, 83% reported that the test was comfortable. In a study with patients with spinal cord injuries 92% of patients reported being comfortable during the measurement, however some reported the duration of time taken to undertake the measurement was unacceptable.^28^ The findings from our small sample contribute to the evidence that indirect calorimetry can be well tolerated by patients, although one patient highlighted the potential limitation of claustrophobia. This study aligns with recent publication by the Global Research Initiative on Post-ICU Nutrition which advocates the need for personalised nutritional strategies with indirect calorimetry cited as a tool to guide this.^28^^,^^29^ In relation to personalised strategies, patients at both sites who received an enhanced nutrition intervention irrespective of whether they received assessment with the indirect calorimeter as it was only one component and interventions were individualised according to patient need. This finding also aligns with the emphasis of ensuring objective and rigorous measurements when monitoring and evaluating patients at risk of malnutrition compared to estimated measures.^30^

Therapeutic relationships with staff were highlighted by patients as an important concept during their recovery. This mirrors previous studies where they are known to lead to improved patient outcomes, and core principles between patients and physiotherapists include being ‘present, receptive, genuine and committed’.^30^ In parallel, factors contributing to meaningful relationships between patients and dietitians in other populations such as those with eating disorders included building trust, being experts and adapting.^31^^,^^32^ Robertson et al., 2024 reported that patients interviewed highlighted the balance of nutritional risk and readiness in treatment approach suggesting proactive approaches to treatments. This balance of caution with proactive progression of rehabilitation and nutritional interventions is likely transferrable to post ICU populations in their desire to recover and return home and could be an area of exploration in the future.

There are several strengths to this research which included the embedding of a qualitative component within a feasibility RCT to provide insights into the acceptability of an intervention mapped according to a framework. This provides a more complete picture of the study feasibility in relation to both quantitative and qualitative data. In addition, the involvement of members from the patient and public involvement and engagement group in the analysis, who reviewed and discussed their personal recovery from periods of critical illness and reported that the themes resonated with them. This contributed to the analytical thinking of the research team given the diversity of the PPIE members past lived experiences. In parallel, the research team drew on their reflexive experience as multi-professionals, with varying years of ICU experience and respective clinical roles to contribute to the analysis development. Multiple round table discussions and iteratives contributed to the development of the final themes. One of the research team who conducted patient interviews, and both who conducted staff interviews were known to participants from the ward environment and previous working relationships. The professional and clinical experience of the research team provided insight into the operational challenges of ward-based rehabilitation and nutrition delivery, supporting empathetic interpretation of participant experiences and the use of appropriate language throughout data collection and analysis.

Several limitations should be noted. The TFA construct of Cost Opportunity was not highlighted by any staff participants preventing a full evaluation of acceptability. It was beyond the scope of these interviews to explore wider of organisational factors, however such considerations and challenges cannot be ruled out as a source of influence on further intervention delivery. As this qualitative evaluation was integrated into a feasibility RCT, the number of patients interviewed were low with data saturation being achieved in this context. Whilst perceptions of tolerance and use of the indirect calorimetry were generally well received, the sample of participants who received assessment from this method was low due to equipment being available on only one study site. Self-selection bias was a risk as patients agreeing to take part may have been more positively disposed towards the intervention. The researchers had sought to seek the experiences of nurses involved in ward care during the intervention delivery period, however due to the diversity of the number of wards patients transferred to, direct nurse interactions in the context of the study participation were low. , In addition, we acknowledge the limited diversity of the participant sample and that perceptions of the intervention may differ across broader patient populations, healthcare settings, and cultural backgrounds. Further research involving larger and more diverse populations would enrich the understanding of how interventions may need adaptation for differing patient needs and contexts.

The delivery of post-ICU rehabilitation and nutritional support is recognised to be affected by organisational pressures, staffing limitations, and variation in ward-based resources. The PHOENIX intervention was therefore designed pragmatically to integrate within existing multidisciplinary ward pathways. A future definitive trial will be important to determine whether enhanced rehabilitation and nutrition interventions improve patient-centred outcomes and downstream healthcare utilisation sufficiently to support wider implementation and healthcare investment.

This study shows that an individualised, structured physiotherapy and optimised nutritional intervention in a ward setting was considered acceptable to both patients and staff. Six of the seven constructs of the TFA were explored with the interviews. The study was regarded as a vehicle to increase awareness and communication of the integrated therapies and recognised the potential of shared educational opportunities between professionals. Indirect calorimetry was well tolerated by patients and facilitated an accurate assessment of nutritional needs. Both patients and staff recognised how these complementary interventions supported physical recovery, and patients reported how therapeutic relationships were perceived as beneficial.

## Contributors

EK: Methodology; Project administration; Data curation; Formal Analysis; Writing—original draft; Writing—review and editing. NW: Methodology; Project administration; Data curation; Formal Analysis; Writing—original draft; Writing—review and editing. DB: Project administration; Data curation; Formal Analysis; Writing—review and editing. ER: Project administration; Data curation; Formal Analysis; Writing—review and editing. HR: Data curation. M N-F: Writing—review and editing. LG Writing—review and editing. ZP: Methodology; Project administration; Writing—review and editing. OG: Methodology; Project administration; Writing—review and editing. DM: Methodology; Project administration; Formal Analysis; Writing—review and editing. EK, NW, DB accessed and verified the underlying data. All authors critically reviewed the manuscript, provided intellectual input, and approved the final version for submission.

## Data sharing statement

Individual participant data underlying the results reported in this article will not be made publicly available because consent for wider data sharing was not obtained from participants at the time of recruitment.

## Declaration of interests

Elizabeth King is Journal co-editor for the Association of Chartered Physiotherapists in Respiratory Care (ACPRC).

Owen Gustafson is funded by the National Institute for Health and Care Research (NIHR) Oxford Biomedical Research Centre (BRC) and Oxford Institute of Applied Health Research. The views expressed are those of the author(s) and not necessarily those of the NIHR or the Department of Health and Social Care.

Zudin Puthucheary is a trustee for the Intenisve Care Society and the National Confidential Enquiry into Patient Outcomes and Death (NCEPOD). He has received grants, consultancy fees, and Honoria payments for lectures from Nestle Health Sciences and grants and consultancy fees from Fresenius Kabi, He is also a named inventor on a ketogenic feed.

The authors have no other conflicts of interest to declare.
