# Supplementary material for: Patient and clinician acceptability of an integrated physiotherapy and nutrition intervention after ICU discharge: a qualitative exploration of a pre-specified co-primary feasibility outcome of the PHOENIX trial
Source: eClinicalMedicine. 2026 Jul 20;98:104085. doi: 10.1016/j.eclinm.2026.104085 (PMC13393731; doi:10.1016/j.eclinm.2026.104085)
Supplement: Supplementary Material 3 [file mmc2.docx]

**PHOENIX study Topic guide – Patient interviews**

**Core themes to explore**

1. Patients’ perceptions of acceptability of the intervention

**QUESTIONS AND PROMPTS**

1. Can you tell me about the rehabilitation you have received with the physiotherapists?
2. What was your experience of receiving rehabilitation with the physiotherapists specifically on the ward after you were discharged from ICU?

Interventions

1. Do you think there been any challenges in your rehabilitation on the ward?

Overcoming these challenges

1. What was your experience regarding the nutritional care you received on the ward?

Method of delivery

1. Do you think there been any challenges with regards to meeting your nutritional needs on the ward?

Overcoming these challenges

Support received

1. What was your experience of having your calorie requirements measured with the indirect calorimeter (if applicable)?
