# Supplementary material for: Patient and clinician acceptability of an integrated physiotherapy and nutrition intervention after ICU discharge: a qualitative exploration of a pre-specified co-primary feasibility outcome of the PHOENIX trial
Source: eClinicalMedicine. 2026 Jul 20;98:104085. doi: 10.1016/j.eclinm.2026.104085 (PMC13393731; doi:10.1016/j.eclinm.2026.104085)
Supplement: Supplementary Material 4 [file mmc3.docx]

**PHOENIX study topic guide - staff**

**Core themes to explore**

1. ‘Provider’ perceptions of acceptability of the intervention

**QUESTIONS AND PROMPTS**

1. Can you tell me about your experience of treating survivors of critical illness to date please?
2. Can you tell me about your experience of supporting patients with their mobilisation following step down to the ward from ICU?

Interventions

1. What do you feel are the challenges of providing mobilisation for patients in the ward after ICU?

Weakness

Lack of available staff or resources to help

Logistics

1. Can you tell me about your experience of supporting patients with their nutrition following step down to the ward from ICU?
2. What do you feel are the benefits or challenges of providing an optimised approach to nutritional care?
3. How could we further improve the implementation of our combined mobilisation and nutrition intervention?

Different interventions

Practicalities of implementation
